# Optimization of Ink Composition and 3D Printing Process to Develop Soy Protein-Based Scaffolds

**DOI:** 10.3390/gels10040223

**Published:** 2024-03-25

**Authors:** Teresa Carranza, Aitor Tejo-Otero, Carlos Bengoechea, Pedro Guerrero, Koro de la Caba

**Affiliations:** 1 BIOMAT Research Group, University of the Basque Country (UPV/EHU), Escuela de Ingeniería de Gipuzkoa, 20018 Donostia-San Sebastián, Spain; teresa.carranza@ehu.eus (T.C.); aitor.tejo@ehu.eus (A.T.-O.); 2 Escuela Politécnica Superior, Universidad de Sevilla, Calle Virgen de África, 7, 41011 Sevilla, Spain; cbengoechea@us.es; 3 Basque Center for Materials (BCMaterials), Applications and Nanostructures, UPV/EHU Science Park, 48940 Leioa, Spain; 4 Proteinmat Materials SL, Avenida de Tolosa 72, 20018 Donostia-San Sebastián, Spain

**Keywords:** bio-based ink, 3D printing, rheology

## Abstract

Inks based on soybean protein isolate (SPI) were developed and their formulations were optimized as a function of the ink heat treatment and the content of other biopolymers to assess the effects of protein–polysaccharides and protein–protein interactions. First, the rheological behavior of the inks was analyzed in relation to the polyvinyl alcohol (PVA) concentration employed (20, 25, and 30 wt%) and, as a result of the analysis, the ink with 25 wt% PVA was selected. Additionally, sodium alginate (SA) and gelatin (GEL) were added to the formulations to improve the viscoelastic properties of the inks and the effect of the SA or GEL concentrations (1, 2, and 3 wt%) was studied. All inks showed shear thinning behavior and self-supporting abilities. Among all the 3D printed scaffolds, those with higher SA (3 wt%) or GEL (2 and 3 wt%) content showed higher shape fidelity and were selected for further characterization. Texture profile analysis demonstrated that the scaffolds prepared with previously heat-treated inks containing 3 wt% GEL showed the highest strength. Additionally, these scaffolds showed a higher water-uptake capacity profile.

## 1. Introduction

In recent years, efforts to formulate tissue engineering materials have shifted from the use of synthetic to natural materials. Although synthetic polymers have excellent mechanical properties and some of them have good biocompatibility, they fail to chemically mimic the cellular microenvironment and do not allow for cell adhesion [1,2], while natural polymers resemble living tissues more closely [2]. Therefore, the combination of natural and synthetic materials has emerged as a way to obtain blends with the required properties. Accordingly, soy protein isolate (SPI) and polyvinyl alcohol (PVA) were used in this work to develop 3D printing inks. PVA, a soluble and biocompatible synthetic polymer [3], was added to SPI formulations to enhance their mechanical properties.

Soy protein, obtained as a by-product from soya oil production, is biocompatible and biodegradable and it has been demonstrated to be a good candidate for tissue engineering due to the presence of RGD-like sequences, which are necessary for promoting stable cell adhesions, and a variety of peptides that promote both cell migration and proliferation [4,5,6,7,8]. As a plant-derived protein, soy protein offers potentially lower immunogenicity compared to animal-derived proteins. In addition, it contains a higher concentration of isoflavones compared to other plant-based proteins, which can influence signaling pathways in eukaryotic cells, particularly by inducing anti-inflammatory activity [9]. Furthermore, SPI has been found to promote adequate cell adhesion and proliferation, accommodating a significant number of human mesenchymal stem cells within small volumes [8]. Accordingly, SPI-based products have been recently assessed for wound healing applications [10]. 

The main components of soy protein are glycinin and β-conglycinin, which can denature and subsequently rearrange themselves, leading to the formation of gel-like structures [11]. Gelation of globular proteins is commonly initiated by heat, which triggers various reactions including protein denaturation or unfolding, dissociation, association, and aggregation. These processes ultimately result in the formation of a three-dimensional network structure [12,13]. Many authors have studied the effect of heat treatments on soy protein-based products, and the results have provided valuable insights for obtaining more stable formulations [14,15,16]. In particular, a temperature of 120 °C has been proposed to obtain films by compression, resulting in flexible and easy-to-handle biocompatible films [17]. As shown by processes which involve temperature changes, such as sterilization [18], the effect of temperature on inks designed for 3D printing still needs to be studied and related back to the properties of the formulated inks.

Additionally, the incorporation of other biopolymers into the SPI ink formulation, such as sodium alginate or gelatin, can be used to promote new physical interactions with the protein and improve its printability and shape fidelity [19,20,21]. In this work, the abovementioned biopolymers were chosen for their abundance, availability, and cost-effectiveness [9,22]. Alginate, a polysaccharide extracted from brown algae, can be used to form hydrogen bonds and electrostatic interactions with the calcium ion that aids the chemical cross-linking of alginate-based printed structures [23]. Regarding gelatin, it is a protein obtained from the hydrolysis of collagen, a structural fibrous protein of the extracellular matrix in animal tissues [24]. Gelatin, unlike collagen, is water-soluble and can be used for the preparation of 3D printing inks [25]. 

The formulation of 3D printing inks requires careful consideration due to the minimum requirements they must fulfil [26]. First and foremost, they must be extrudable, which requires flow properties under shear. In addition, they must maintain dimensional stability after deposition to ensure fidelity to the intended printed object. These properties can be achieved through various strategies, such as the use of thermosensitive materials or materials able to be chemically or physically cross-linked. 

In this work, 3D printing inks based on SPI, with the inclusion of PVA to improve the mechanical behavior of the 3D printed scaffolds, were investigated in terms of their composition. Specifically, their suitability for blending with a protein (gelatin) or with a polysaccharide (alginate), as well as the effect of temperature, were evaluated through printability analyses. These polymers were used to formulate 3D printing inks suitable for the production of high-resolution structures.

## 2. Results

### 2.1. Rheological Behavior of 3D Printing Inks

**Figure 1:**
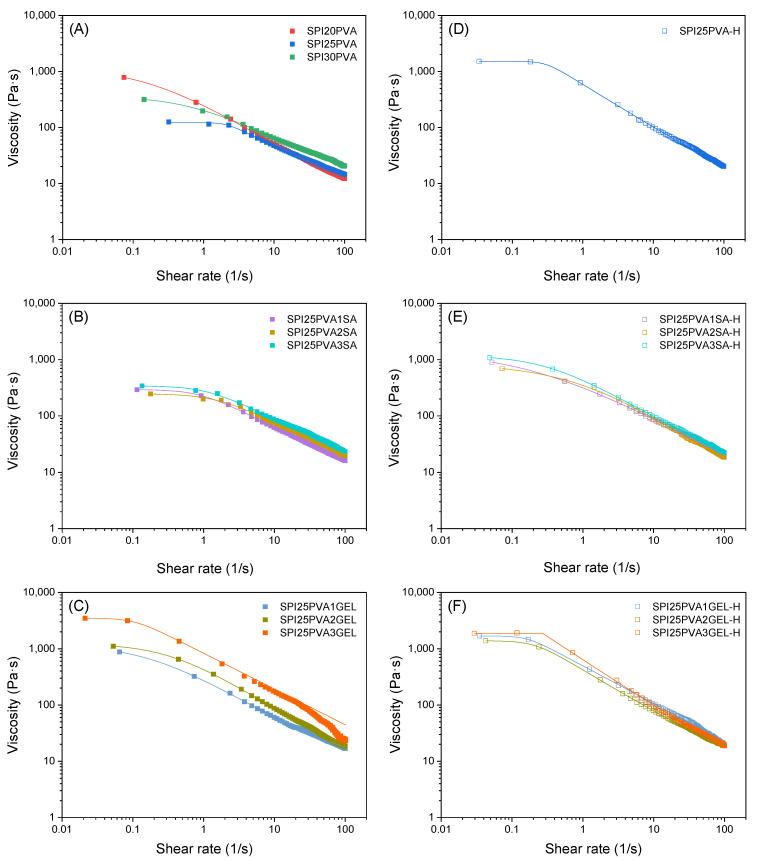
Viscosity curves of SPI inks with different contents of PVA (20, 25, and 30 wt%) before (**A**) and after heat treatment (**D**). Viscosity curves of SPI25PVA ink with different contents of SA (1, 2, and 3 wt%) before (**B**) and after heat treatment (**E**). Viscosity curves of SPI25PVA ink with different contents of GEL (1, 2, and 3 wt%) before (**C**) and after heat treatment (**F**). All the tests were performed at 30 °C. Lines represent the fitting to Carreau–Yasuda model.

To enable processing, inks used in 3D printing must demonstrate appropriate rheological properties, exhibiting shear thinning behavior whereby viscosity reduces with an increasing shear rate [26]. This property is crucial for smooth ink flow through the nozzle without obstruction, facilitated by the narrowest tip section where maximum shear occurs, calculated using the Weissenberg–Rabinowitsch–Mooney Equation (2). To evaluate the suitability of the ink formulations for 3D printing, shear flow tests were performed at 30 °C to simulate the material behavior at the 3D printer head and, thus, during the 3D printing process. The results depicted in Figure 1 indicate that the viscosity decreased with increasing shear rate for all samples, demonstrating shear thinning behavior, which is desirable for 3D printing as it allows the ink to flow when subjected to specific shear stress [26]. Data were fitted to the Carreau–Yasuda model, which showed that all *n* values were below 1 (Table S1), confirming the shear thinning behavior of the inks [27]. At low shear rates, the inks demonstrated Newtonian behavior, as seen in the initial viscosity values, and the negative slope was dependent on the *a* and *n* parameters. The data presented in Figure 1A show that the samples with varying PVA concentrations had similar slopes, suggesting similar flowability characteristics. Notably, the SPI25PVA ink exhibited the lowest values for viscosity (both *η*_0_ and *η*_∞_), indicating higher extrudability. Moreover, increasing the PVA concentration to 30 wt% did not improve the flow properties of soy protein. Consequently, higher concentrations were discarded for this reason. Based on these findings, the ink formulation containing 25 wt% PVA was selected for further investigation, specifically to evaluate the impact of temperature and the addition of SA or GEL.

The incorporation of SA resulted in slightly higher viscosity values, with no significant differences among samples, as shown in Figure 1B. In contrast, the incorporation of GEL led to a noticeable increase in viscosity, particularly in the highest GEL content under study, as depicted in Figure 1C. This viscosity increase suggests the occurrence of new interactions, such as hydrogen bonds, between the polar groups of the biopolymers present in the ink formulation. This phenomenon can be attributed to the greater range of functional groups present in gelatin in comparison to sodium alginate. In particular, amino, carboxyl, hydroxyl, or thiol groups in gelatin can be engaged in hydrogen bonding with SPI [22]. As for sodium alginate, it has been reported that this polysaccharide can form electrostatic interactions with globular proteins and establish complexes due to its net negative charge at neutral pH [28]. Additionally, it has been shown that sodium alginate can be engaged in hydrogen bonding interactions [29]. However, it is worth noting that the protein –polysaccharide interaction effect is less prominent compared to the protein–protein interactions observed in the formulations with gelatin. 

Since temperature can promote protein denaturation or unfolding, facilitating interactions among the components of the formulation [12,13], this effect was analyzed as an strategy to improve the properties of the resulting 3D printed structures. Regarding the effect of the heat treatment of the inks, the SPI25PVA-H formulation exhibited a higher η_0_ value compared to SPI25PVA, as shown in Figure 1D and Table S1. Additionally, the heat treatment of the ink resulted in the onset of the shear-thinning region taking place at lower shear rates, which can be attributed to protein unfolding [14] facilitating physical interactions between the polar groups of SPI and the hydroxyl groups of PVA [30]. In fact, temperature has been shown to have an effect of exposing reactive groups that can lead to cross-linking or rearrangement of the protein structure [28,31]. To assess the effect of temperature, SDS–PAGE was performed on the SPI25PVA and SPI25PVA-H gels. SDS was applied to disrupt hydrogen bonds as well as electrostatic and hydrophobic interactions, while β-ME was used to break S–S linkages [20]. As depicted in Figure S1, all samples showed the major subunits of soy protein isolate: glycinin (11 S), which has two subunits, an acidic (AS) and a basic (BS) subunit; and β-conglycinin (7 S), a glycoprotein that forms a trimer consisting of α, α′, and β subunits, which can display associative and dissociative phenomena [21,32]. No band shift was observed between samples without reduction treatment (lines 1, 2 and 5, 6) and reduced samples (lines 3, 4 and 7, 8). Based on these results, it can be deduced that there were no chemical reactions between soy protein and polyvinyl alcohol, as no bands with different molecular weights were observed between heat-treated and non-heat-treated inks for both reduced and non-reduced samples. Similarly, Chen et al. [20] demonstrated that sodium alginate and gelatin did not chemically interact with soy protein. Therefore, any possible interactions that may be observed in these systems may be due to physical interactions. 

A similar temperature effect was observed in the systems containing SA (Figure 1E) or GEL (Figure 1F). This phenomenon is consistent with the findings of Garrido et al., who reported that a higher temperature during processing led to a higher degree of denaturation of SPI, promoting physical cross-linking [33]. 

**Figure 2:**
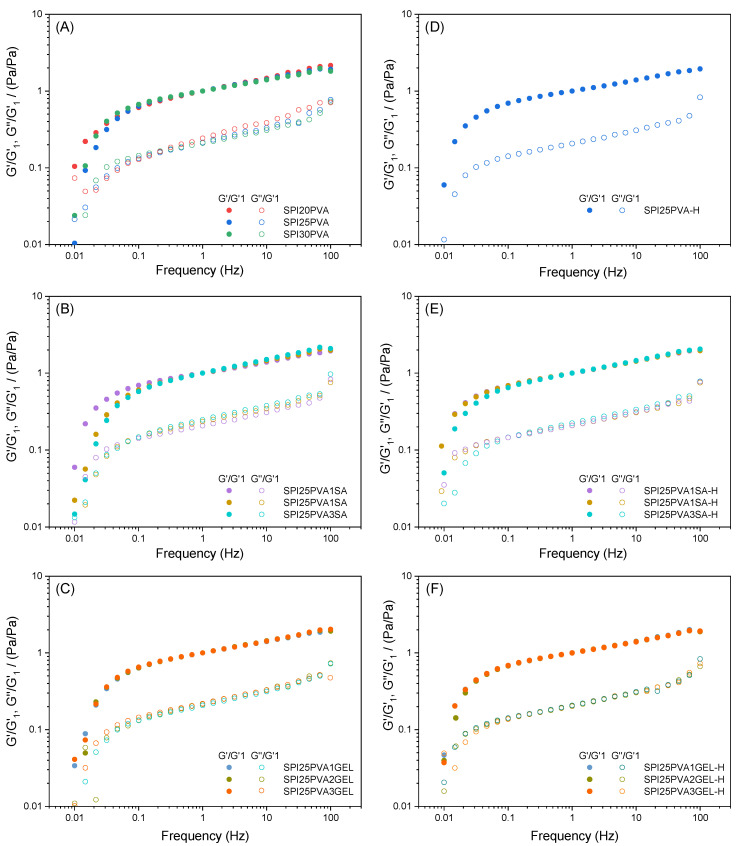
Normalized frequency sweeps of SPI inks with different contents of PVA (20, 25, and 30 wt%) before (**A**) and after heat treatment (**D**). Normalized frequency sweeps of SPI25PVA ink with different contents of SA (1, 2, and 3 wt%) before (**B**) and after heat treatment (**E**). Normalized frequency sweeps of SPI25PVA ink with different contents of GEL (1, 2, and 3 wt%) before (**C**) and after heat treatment (**F**). The corresponding elastic moduli at 1 Hz (G′_1_) are used as normalization factors for both G′ and G″.

After assessing the suitability of the inks to be extruded, their self-supporting abilities were analyzed through frequency sweeps (Figure 2). All mechanical spectra displayed similar qualitative behaviors when comparing samples where a specific parameter is studied, such as PVA, SA, or GEL content or the effect of heating. Thus, when plotting the normalized mechanical spectra, using a value for the elastic modulus of 1 Hz (G′_1_) as the normalization factor, this similar qualitative behavior can be observed in Figure 2. The overlap was especially good for systems with gelatin, either before or after heating (Figure 2C,F, respectively). Concerning systems with just PVA or with SA, the systems with the lowest amount of the biopolymer always showed a slight deviation (SPI20PVA, SPI25PVA1SA). 

**Figure 3:**
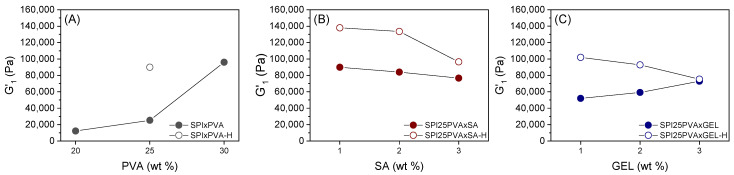
Evolution of the elastic moduli of SPI inks at 1 Hz (G′_1_) with different contents of PVA (20, 25, and 30 wt%) and the system with 25 wt% PVA after heat treatment (**A**); SPI25PVA inks with different contents of SA (1, 2, and 3 wt%) before (dashed line) and after heat treatment (solid line) (**B**); and SPI25PVA inks with different contents of GEL (1, 2, and 3 wt%) before (dashed line) and after heat treatment (solid line) (**C**).

While Figure 2 shows the normalized mechanical spectra (viscoelastic moduli divided by their corresponding G′ at 1 Hz) to check the similarities between their viscoelastic behavior independently of their formulation, the values in Figure 3 show their actual elastic moduli (without being divided by G′ at 1 Hz). Considering the evolution of G′_1_ in formulations with PVA (Figure 3A), it may be observed that an increase in the viscoelastic moduli of around 2 or almost 8 times its original value results from increasing the PVA content from 20 to 25 or 30%, respectively. However, the effect of SA is not so apparent (Figure 3B), as G′_1_ remained around 80,000 ± 4000 Pa for all systems (SPI25PVA1SA, SPI25PVA2SA, SPI25PVA3SA). In systems with GEL (Figure 3C), a more discrete upwards evolution of G′_1_ may be observed (its value being 1.1 and 1.4 times higher for SPI25PVA2GEL and SPI25PVA3GEL, respectively, as compared to SPI25PVA1GEL). When samples were heated, all samples strengthened when compared to their corresponding unheated counterparts, especially SPI25PVA-H. When either SA or GEL were added into the formulation, this strengthening was lesser when a higher percentage was included in the formulation, and also showed a decrease as the percentage of either SA or GEL increased.

When the ratio between the loss modulus (G″) and the storage modulus (G′), expressed as the loss tangent (tan δ), are analyzed, tan δ values were found to be lower than 1 (Figure S2), indicating that the loss modulus (G″) was smaller than the storage modulus (G′) and suggesting that the inks exhibited solid-like behavior [20,34]. The inks with different concentrations of PVA (Figure S2A) showed a similar trend in tan δ throughout the frequency sweep, with values around 0.25, as did the SPI25PVA-H inks (Figure S2D), suggesting that the heat treatment did not exert a major influence on the viscoelasticity of the inks. The inks with SA (Figure S2B) or GEL (Figure S2C) exhibited similar values to the samples without these biopolymers, with higher values, especially at lower frequencies, for the inks with higher SA or GEL content. This trend was also observed for heat-treated inks with SA (Figure S2E) or GEL (Figure S2E). As reported by Dick et al. [34], these values of tan δ between 0.20 and 0.30 suggest good stability in 3D printed structures. When the values of tan δ at 1 Hz (tan δ_1_) are examined, a greater content of SA leads to more a viscous character (higher tan δ values). For GEL systems, this is not so apparent. In all cases, heating leads to a strengthening which can be deduced by lower tan δ values.

**Figure 4:**
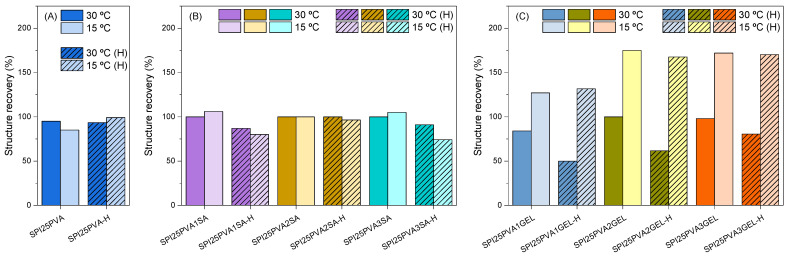
Structure recovery percentage for (**A**) SPI25PVA inks with (**B**) different contents of SA (1, 2, and 3 wt%) and (**C**) different contents of GEL (1, 2, and 3 wt%) as a function of test temperature (dark color, 30 °C; light color, 15 °C). Line patterns are used for heat-treated samples (designated with an H at the end of the name). An example of the raw data used to calculate the values is shown in Figure S3.

Additionally, three interval thixotropic testing was carried out to simulate any changes that may occur during the extrusion process and then assess the self-supporting abilities of the inks (Figure 4). For that, the temperatures selected were 30 °C, the 3D printing temperature, and 15 °C, the temperature on the 3D printer platform. The thixotropic test is based on the viscosity recovery measurement after applying a specific shear, calculated by equation (2) using data from the preliminary printing tests and Carreau–Yasuda fitting. The purpose of this test is to simulate the 3D printed ink deposition on the platform. Figure 4A depicts the recovery percentage of samples containing 25 wt% PVA, wherein no significant effect of the test temperature (15 or 30 °C) or ink heat-treatment was observed. The same behavior can be seen in Figure 4B for the inks with alginate, wherein the test temperature did not cause any relevant difference in the recovery percentages; however, these recovery percentages were slightly lower for heat-treated inks. Regarding the inks prepared with different GEL contents (Figure 4C), these samples showed different recovery performances as a function of the test temperature; in particular, inks showed higher recovery percentages at 15 °C. This behavior can be explained by the thermal reversibility of gelatin in forming intramolecular hydrogen bonds, leading to the gelation process [19,35,36]. As for the heat-treated samples, the most noticeable change was observed for the heat-treated inks with GEL, which showed a lower recovery capacity compared to non-heat-treated samples when the test temperature used was 30 °C. This can be attributed to the higher movement of gelatin chains, which are able to interact with SPI chains, decreasing the recovery capacity of SPI inks [20,37]. 

### 2.2. Shape Fidelity of 3D Printed Scaffolds 

**Figure 5:**
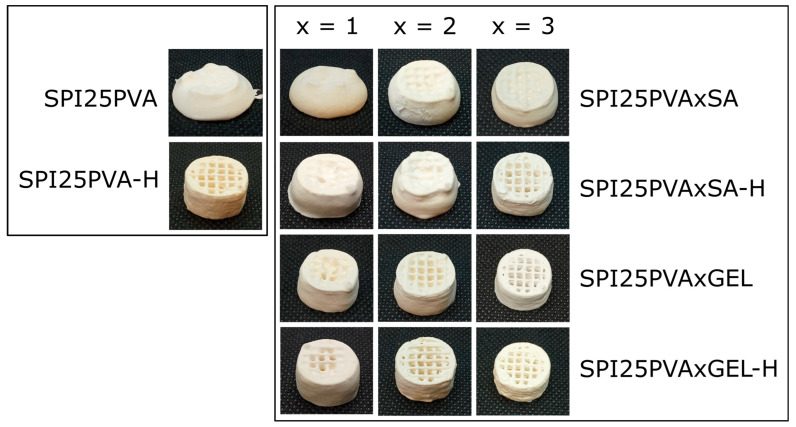
Photographs of SPI 3D printed scaffolds with polyvinyl alcohol (25 wt% PVA) as a function of sodium alginate (1, 2, or 3 wt% SA) or gelatin (1, 2, or 3 wt% GEL) contents and heat treatment (H).

All the samples could be printed, as demonstrated by a Pr index around 1.0 (Figure S4). A printability value of 1 implies that the pore is completely square, as expected, while values further away from this denote less definition. A confidence margin of 5% has been used, as marked in Figure S4, to observe those closest to the desired value. As can be seen in Figure 5, GEL-containing specimens showed greater pore definition than those with SA. Specifically, scaffolds with higher SA (3 wt%) or GEL (2 and 3 wt%) content, as well as SPI25PVA-H samples, presented better shape fidelity, and no pore-clogging was observed. It can be deduced that the heat treatment further rearranges SPI, allowing for greater levels of entanglement [14,15]. These results are in accordance with those obtained by the printability test (Figure S4), which showed that the heat treatment of the inks decreased the dispersion of the Pr index values. Since the analysis of 3D printed scaffolds revealed that non-heat-treated control samples and those with 1 wt% SA, 2 wt% SA, and 1 wt% GEL (regardless of heat treatment) collapsed as the printed structure gained height, those formulations were discarded for further assessments.

The differences obtained in the scaffolds with alginate or gelatin can be attributed to the different interactions of soy protein with sodium alginate or gelatin. The most relevant difference lies in the nature of the protein–protein and protein–polysaccharide interactions. On the one hand, gelatin, with its broad range of functional groups, facilitates the occurrence of physical interactions with SPI [37]. On the other hand, sodium alginate only possesses hydroxyl and carboxyl groups, so the entanglement may be less significant [22]. This could be the reason behind the strengthening observed as the GEL content increased in the unheated systems, which was not observed for SA systems, in the rheological tests.

### 2.3. Mechanical Properties of 3D Printed Scaffolds

3D printed scaffolds were analyzed using textural profile analysis, which consisted of two repetitions of uniaxial compression at a 20% strain value. This analysis, commonly used for food-related applications, is gaining increasing significance in the field of tissue engineering [38,39,40,41]. In this analysis, hardness is the maximum force that occurred at the first compression and is related to the stability and strength of the scaffolds [39]. As can be seen in Table 1, the values of hardness for the scaffolds with SA were lower than those found in the SPI25PVA-H sample, while hardness was higher in the scaffolds with GEL, especially in with 3 wt% GEL whose inks had been previously heat-treated. A similar trend was observed in the study conducted by Sun et al., where mild heat conditions resulted in increased hardness values [42]. These results can be related to those obtained in these 3D printing tests, suggesting that the scaffolds with the highest shape-fidelity are those that show the highest hardness values. 

Table 1 also shows cohesiveness values, which refer to the ability of a scaffold to withstand a second deformation concerning its resistance under the first deformation. This provides information about elastic recovery, which is related to the extent to which a material can return to its original shape after being deformed and released. Therefore, this analysis of cohesiveness is crucial for understanding the elastic behavior of 3D printed scaffolds. When the cohesiveness is very close to one, it indicates that the scaffold recovers its initial shape and size with behavior similar to that of rubber [43]. The cohesiveness values of all samples ranged from 0.70 to 0.84, with SPI25PVA3GEL-H samples showing the lowest cohesiveness value around 0.56. This result is consistent with the hardness values measured, indicating that the heat-treated scaffolds with higher gelatin concentrations exhibited stiffer behavior compared to the others, with lower recovery capacity but higher strength. 

### 2.4. Water Uptake of 3D Printed Scaffolds

**Figure 6:**
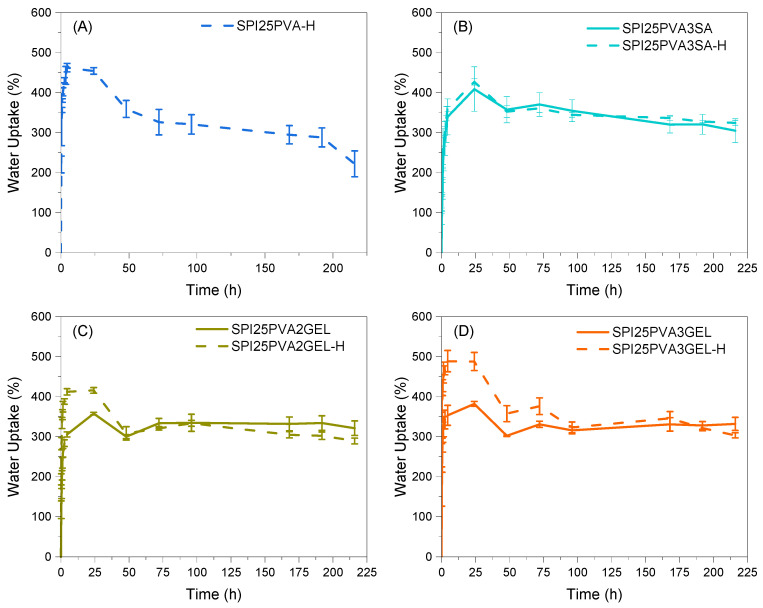
Water uptake values for SPI25PVA-H (**A**), SPI25PVA3SA and SPI25PVA3SA-H (**B**), SPI25PVA2GEL and SPI25PVA2GEL-H (**C**), and SPI25PVA3GEL and SPI25PVA3GEL-H (**D**) scaffolds.

The water uptake behavior of the developed scaffolds was analyzed, since the scaffold water uptake capacity is considered essential for tissue engineering applications due to the need to transport body fluids with nutrients to cells [21,44]. Importantly, all scaffolds maintained their structural integrity after immersion for 200 h. As can be seen in Figure 6, all the scaffolds reached an equilibrium water uptake of 300%, showing a capacity to absorb water and ensure the transport of nutrients inside the 3D printed scaffold. As shown in Figure 6A, the SPI25PVA-H scaffold exhibited its maximum water uptake capacity within the first hour, reaching nearly 500%. After 100–125 h, these values stabilized and a decrease in the water uptake percentage, related to the degradation of the material itself, started to be observed. In contrast, the scaffolds with 3 wt% SA (Figure 6B) showed a slightly lower maximum absorption, with no significant difference between heat-treated and non-heat-treated scaffolds. The scaffolds containing GEL (Figure 6C,D) displayed a higher initial water uptake capacity when the inks had been heat-treated (Figure 6C,D); this effect was more noticeable at higher GEL contents (Figure 6D). The addition of SA and GEL was observed as beneficial to improve water holding capacity over time, since the swelling capacity of all the samples is directly related to the presence of polar groups within the scaffolds [45]. This effect could be due to the new physical interactions formed between SPI and SA or GEL [46,47].

## 3. Conclusions

Soy protein-based inks exhibited shear thinning behavior during extrusion 3D printing and demonstrated self-supporting capabilities after 3D printing. The addition of alginate or gelatin, along with heat treatment, were proved to be beneficial to improving printability. No chemical reaction was observed between SPI and PVA due to heat treatment, as confirmed by electrophoresis. Furthermore, the interactions between SA and SPI were found to be weaker compared to those between GEL and SPI, which can be attributed to the nature of the molecules, with protein–protein interactions being stronger than polysaccharide–protein interactions due to the higher diversity of functional groups in proteins. Among the samples, the SPI25PVA3GEL-H inks exhibited the best performance in the rheological tests. Additionally, texture profile analysis revealed that 3D printed SPI25PVA3GEL-H samples exhibited the highest hardness values. As for the water uptake assays, both gelatin and alginate improved the water-holding capacity over time. Overall, these findings highlight the potential of plant-based protein systems for developing suitable inks that can be 3D printed to produce self-supporting structures. Although the polymers used in the ink formulation could indicate their viability for demanding applications, such as tissue engineering, further analyses should be carried out to assert the potential of the inks and 3D printed structures developed in this work in order to be used for biomedical applications. 

## 4. Materials and Methods

### 4.1. Materials

Soy protein isolate (SPI), with 90% protein on a dry basis, was supplied by ADM Protein Specialties Division (Amsterdam, The Netherlands); SPI has 5% moisture, 4% fat, and 5% ash and its isoelectric point is 4.6 [17]. Polyvinyl alcohol (PVA) (89,000–98,000 molecular weight, 99% hydrolyzed) and phosphate buffered saline (PBS) tablets were purchased from Sigma-Aldrich (Madrid, Spain). Porcine gelatin (GEL), with a bloom of 270, was supplied by Sancho de Borja (Borja, Spain). Sodium alginate (SA), with a viscosity of 350–550 mPa·s (1%, 20 °C), and glycerol (Gly), used as plasticizer, were supplied by PanReac (Barcelona, Spain). 

### 4.2. Ink Preparation

On the one hand, soy protein isolate (SPI) was manually mixed with 20 wt% glycerol (based on SPI’s dry basis). On the other hand, three polyvinyl alcohol (PVA) solutions were prepared by dissolving PVA (20, 25, and 30 wt%, based on SPI’s dry basis) in type II miliQ water under magnetic stirring at 120 °C. After that, the SPI mixture was added to the PVA solution and manually mixed to obtain a SPI concentration of 25 *w*/*v*%, and the resulting gels were denoted as SPI20PVA, SPI25PVA, and SPI30PVA, as a function of the PVA content. 

From the results of the first part of the study, the SPI25PVA formulation was selected as the most suitable gel for further investigation. This gel was mixed at 60 °C with SA or GEL solutions to obtain a SA or GEL concentration of 1, 2, or 3 wt% (based on SPI’s dry basis). Higher concentrations of GEL or SA were not studied due to processing difficulties. The resulting gels were designated as SPI25PVA1SA, SPI25PVA2SA, and SPI25PVA3SA, based on the concentration of SA, or as SPI25PVA1GEL, SPI25PVA2GEL, and SPI25PVA3GEL, based on the concentration of GEL. The designations of the inks are summarized in Table 2. 

Additionally, SPI gels with 25 wt% PVA were heated at 120 °C for 20 min to analyze the effect of temperature on the ink printability and on scaffold properties. Those samples were designated with an H at the end of the name, as specified in Table 2. 

### 4.3. Rheological Evaluation

The rheological assessment was carried out with a Thermo Scientific Haake RheoStress1 rheometer (IFI, Vigo, Spain), equipped with a 35 mm diameter serrated plate–plate configuration. A gap of 1 mm was used for all measurements. First, linear viscoelastic region (LVR) tests were conducted at 30 °C to determine the most suitable strain for subsequent oscillatory tests. Then, frequency sweeps were carried out from 0.05 to 100 Hz at 30 °C and a constant strain value of 0.01 within the LVR.

Shear flow tests were performed from 0.01 to 100 s^−1^ shear values at 30 °C. All sweeps were carried out at least in triplicate and mean curves were represented. Flow curves were fitted using the Carreau–Yasuda rheological model, which describes pseudoplastic flow (1) [27]:

(1) $$\eta \;(\dot{\gamma })=\eta_{\infty }+\left(\eta_{0}-\eta_{\infty }\right){\left[1+{\left(\lambda_{c}\dot{\gamma }\right)}^{a}\right]}^{\left(\frac{n-1}{a}\right)}$$

where η is viscosity, η_∞_ and η_0_ are viscosity at infinite and zero limits, λ_c_ is the relaxation time constant, ẏ is the shear rate, n is the power law index or flow index, and a is the transition control factor or Yasuda exponent. 

To simulate the effect of ink extrusion and deposition, three interval thixotropy tests were performed using 75 s^−1^ (average value obtained by Equation (2)) as a high shear value and 0.1 s^−1^ as a low shear value. Tests were performed at 30 °C to simulate the extrusion process and recovery percentages were calculated at 30 °C and also at 15 °C to simulate 3D printing platform conditions. Shear values on the nozzle wall (ẏ_w_) for each printing condition were calculated using the Weissenberg–Rabinowitsch–Mooney Equation (2) [48].

(2) $${\dot{\gamma }}_{w}=\frac{4Q}{\pi r^{3}}\left(\frac{3n+1}{4n}\right)$$

where Q is the volumetric flow rate, r is the radius of the tip, and n is the power law index or flow index obtained from the rheological model (Table S1). Volumetric flow values were obtained considering filament length (equivalent diameter for a 10 mL syringe) and printing times given by Cura 5.3.1 slicing software (Ultimaker, Geldermalsen, The Netherlands). 

Finally, the structure recovery percentage was calculated using Equation (3).

(3) $$\mathrm{Structure}\;\mathrm{recovery}\;\left(\%\right)=\frac{\eta_{2}}{\eta_{1}}100$$

where η_1_ and η_2_ represent the average viscosity value at 0.1 s^−1^ before and after applying the extrusion shear consecutively.

### 4.4. Sodium Dodecyl Sulphate–Polyacrylamide Gel Electrophoresis (SDS–PAGE)

SPI25PVA and SPI25PVA-H gels were tested under non-reducing conditions and under reducing conditions using β-mercaptoethanol (β-ME). SDS–PAGE electrophoresis was carried out using Laemmli’s method with 12% separating gel and 8% stacking gel [49]. Protein separation was performed using a Mini-PROTEAN tetra cell electrophoresis apparatus (Bio-Rad, Madrid, Spain), and protein bands were visualized using Bio-Safe Coomassie staining. Precision Plus Protein^TM^ standards (Bio-Rad, Spain) were used as a standard reference. Two wells per sample were loaded.

### 4.5. 3D Printing

3D printing tests were carried out using a domoBIO 2A printer equipped with a 10 mL luer-lock syringe extruder and refrigerated platform with a porta adapter (Domotek, Tolosa, Spain). The scaffold designed was a 21 mm diameter and 10 mm height cylinder. However, to determine the printability of the inks, the test specimen was designed as a 21 mm diameter and 1 mm height cylinder. Both models were designed using Solid Edge (Siemens, Munich, Germany) CAD software and they were sliced separately using Cura 5.3.1 slicing software (Ultimaker, Geldermalsen, The Netherlands). All the specimens were printed using 16 G (1.19 mm inner diameter) plastic conical tips at a 15 mm/s printing speed. Layer height was set up at 0.3 mm, line width at 1.2 mm, and infill line distance at 2.5 mm. The flow rate was optimized for each sample and turned out to be between 160 and 190% (0.41 and 0.49 mL/min). Nozzle temperature was established at 30 °C, whereas platform temperature was set up at 15 °C for the formulations with GEL and left at room temperature for the rest of the formulations. 

Printability (Pr) was determined based on the circularity (C) of the area enclosed by the grid holes, using Equations (4) and (5) for determining both parameters [50]:

(4) $$\mathrm{Pr}=\frac{\pi }{4}\frac{1}{C}$$

(5) $$C=\frac{4\pi A}{L^{2}}$$

where L is the perimeter of the infill pattern square and A is the area of the infill pattern calculated using Image J 1.54i software [51]. The value of C is 1 when the shape is a perfect circle, while it is π/4 when it is a square shape. A perfect print structure would have a Pr value of 1, while one with circular grid holes will have Pr values lower than 1.

### 4.6. Mechanical Properties 

TA.XT plusC Texture Analyzer (Aname Instrumentación Científica, Madrid, Spain), equipped with a 5 kg load cell, was used to carry out texture profile analysis. Tests were performed at a 20% strain value with a 50 mm aluminium cylinder (P/50) and a crosshead speed of 1 mm/s. All samples were conditioned in a controlled chamber at 23 °C and 100% relative humidity before testing. Four replicates for each sample were analyzed. Data were collected and assessed using Exponent Connect 8.0.16.0 Lite software (Stable Micro Systems, Godalming, UK).

### 4.7. Water Uptake (WU)

A water uptake test was carried out using PBS media (pH 7.4). Samples were weighed (*W*_0_), immersed into 40 mL of water at 37 °C, taken out of the medium at specific times, and weighed again (*W_t_*) to determine water uptake using Equation (6) [52]: 


(6) $$WU\;\left(\%\right)=\frac{W_{t}-W_{0}}{W_{0}}\;100$$

### 4.8. Statistical Analysis

Data were analyzed using a one-way analysis of variance (ANOVA) performed with the SPSS Statistics 25.0 software program (IBM, Armonk, NY, USA). Subsequently, multiple post-hoc comparisons were carried out using Tukey’s test with a significance level of *p* ≤ 0.05.

## Figures and Tables

**Table 1:** Hardness and cohesiveness values obtained by texture profile analysis. The mean comparison was done for each column separately (Hardness and Cohesiveness) according to Tukey’s multiple comparison test; different letters (a–c) reveal significant differences (*p* ≤ 0.05).

Sample	Hardness (g)	Cohesiveness
SPI25PVA-H	2923 ± 139 ^a^	0.704 ± 0.184 ^a^
SPI25PVA3SA	2027 ± 51 ^ab^	0.843 ± 0.002 ^a^
SPI25PVA3SA-H	2170 ± 61 ^b^	0.822 ± 0.003 ^a^
SPI25PVA2GEL	3001 ± 121 ^b^	0.830 ± 0.003 ^ab^
SPI25PVA2GEL-H	3008 ± 168 ^b^	0.830 ± 0.001 ^ab^
SPI25PVA3GEL	3402 ± 70 ^c^	0.700 ± 0.085 ^ab^
SPI25PVA3GEL-H	3761 ± 361 ^c^	0.564 ± 0.050 ^b^

**Table 2:** Ink designation as a function of composition.

Ink designation	SPI (*w*/*v*%)	PVA (wt%)	SA (wt%)	GEL (wt%)
SPI20PVA	25	20	0	0
SPI25PVA	25	25	0	0
SPI30PVA	25	30	0	0
SPI25PVA1SA	25	25	1	0
SPI25PVA2SA	25	25	2	0
SPI25PVA3SA	25	25	3	0
SPI25PVA1GEL	25	25	0	1
SPI25PVA2GEL	25	25	0	2
SPI25PVA3GEL	25	25	0	3

## Data Availability

Data will be made available on request.

## Supplementary Materials

The following supporting information can be downloaded at https://www.mdpi.com/article/10.3390/gels10040223/s1, Table S1. Parameters of the Carreau-Yasuda rheological model and shear rate value in the printing tip wall for all samples as a function of PVA, SA and GEL and heat treatment (H); Figure S1. SDS-PAGE electrophoresis of SPI25PVA (lines 1 and 2), SPI25PVA reduced by β-ME (lines 3 and 4), SPI25PVA-H (lines 5 and 6), and SPI25PVA-H reduced by β-ME (lines 7 and 8); Figure S2. Frequency sweeps of SPI inks with different contents of PVA (20, 25 and 30 wt %) before (A) and after heat treatment (D). Frequency sweeps of SPI25PVA ink with different contents of SA (1, 2, and 3 wt %) before (B) and after heat treatment (E). Frequency sweeps of SPI25PVA ink with different contents of GEL (1, 2, and 3 wt %) before (C) and after heat treatment (F); Figure S3. Three interval thixotropy test results for SPI25PVA1GEL ink at 30 °C recovery temperature; Figure S4. Printability index for SPI25PVA inks with A) different contents of SA (1, 2, and 3 wt %) and B) different contents of GEL (1, 2, and 3 wt %).
