# Supplementary material for: Optimization of Ink Composition and 3D Printing Process to Develop Soy Protein-Based Scaffolds
Source: Gels. 2024 Mar 25;10(4):223. doi: 10.3390/gels10040223 (PMC11049190; doi:10.3390/gels10040223)
Supplement: Supplementary file 1 [file gels-10-00223-s001.zip › gels-2916469-supplementary.pdf]

Supplementary Material

Table S1. Parameters of the Carreau-Yasuda rheological model and shear rate value in the printing tip wall for all samples as a function of PVA, SA and GEL and heat treatment (H).

Sample	η_0 (Pa·s)	η_∞ (Pa·s)	λ_c (s)	a	n	R^2	$\dot{\gamma}_w$ (s^{-1})
SPI20PVA	1190.5	4.9	5.5	0.8	0.20	0.99	-
SPI25PVA	121.8	4.5	0.5	5.3	0.37	0.99	68
SPI25PVA-H	1518.0	5.6	3.4	4.3	0.22	0.99	91
SPI30PVA	365.0	8.2	1.5	0.9	0.34	0.99	-
SPI25PVA1SA	294.1	4.0	1.1	1.9	0.33	0.99	72
SPI25PVA1SA-H	1458.0	1.0	5.0	0.6	0.31	0.99	75
SPI25PVA2SA	294.1	4.0	1.1	1.9	0.33	0.99	72
SPI25PVA2SA-H	860.6	8.0	0.7	0.6	0.28	0.99	79
SPI25PVA3SA	341.3	2.5	1.0	1.8	0.40	0.99	66
SPI25PVA3SA-H	1187.4	5.2	3.3	1.1	0.28	0.99	79
SPI25PVA1GEL	1208.9	8.7	5.8	0.9	0.24	0.99	87
SPI25PVA1GEL-H	1688.1	0.0	6.2	3.6	0.33	0.99	73
SPI25PVA2GEL	1223.1	3.6	3.1	1.1	0.23	0.99	88
SPI25PVA2GEL-H	1387.5	7.2	4.8	2.7	0.24	0.99	87
SPI25PVA3GEL	3499.9	0.0	9.6	2.8	0.36	0.99	69
SPI25PVA3GEL-H	1870.5	5.8	3.7	55.0	0.17	0.99	107

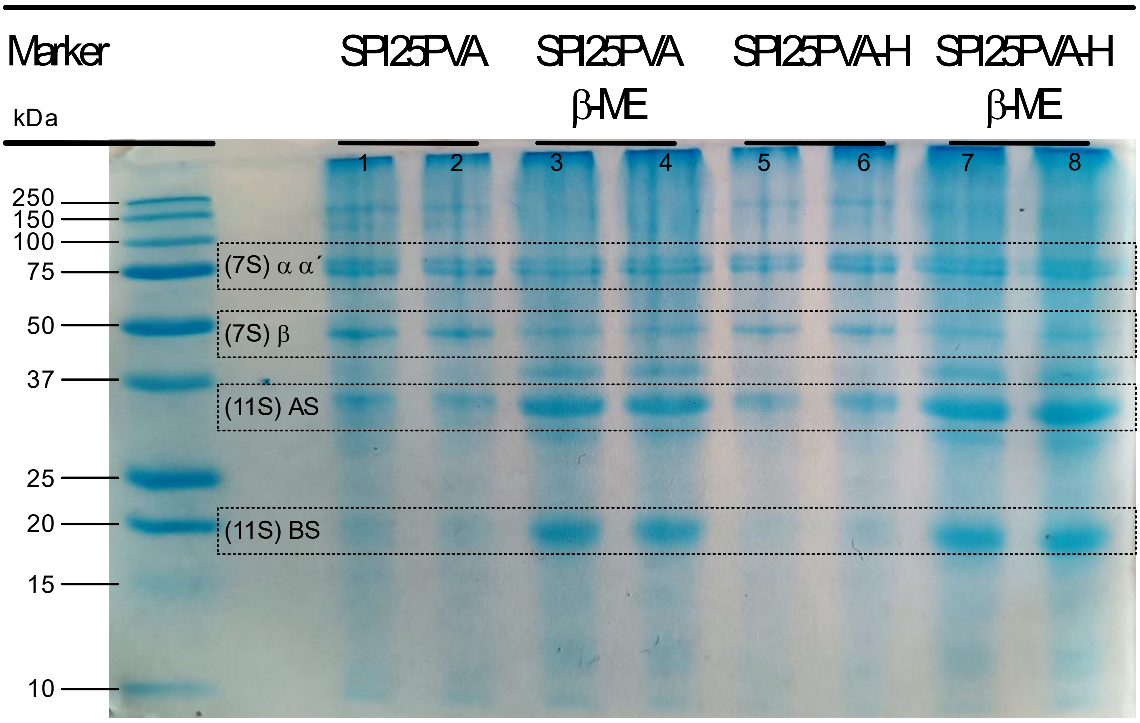

Figure S1. SDS-PAGE electrophoresis of SPI25PVA (lines 1 and 2), SPI25PVA reduced by β-ME (lines 3 and 4), SPI25PVA-H (lines 5 and 6), and SPI25PVA-H reduced by β-ME (lines 7 and 8).

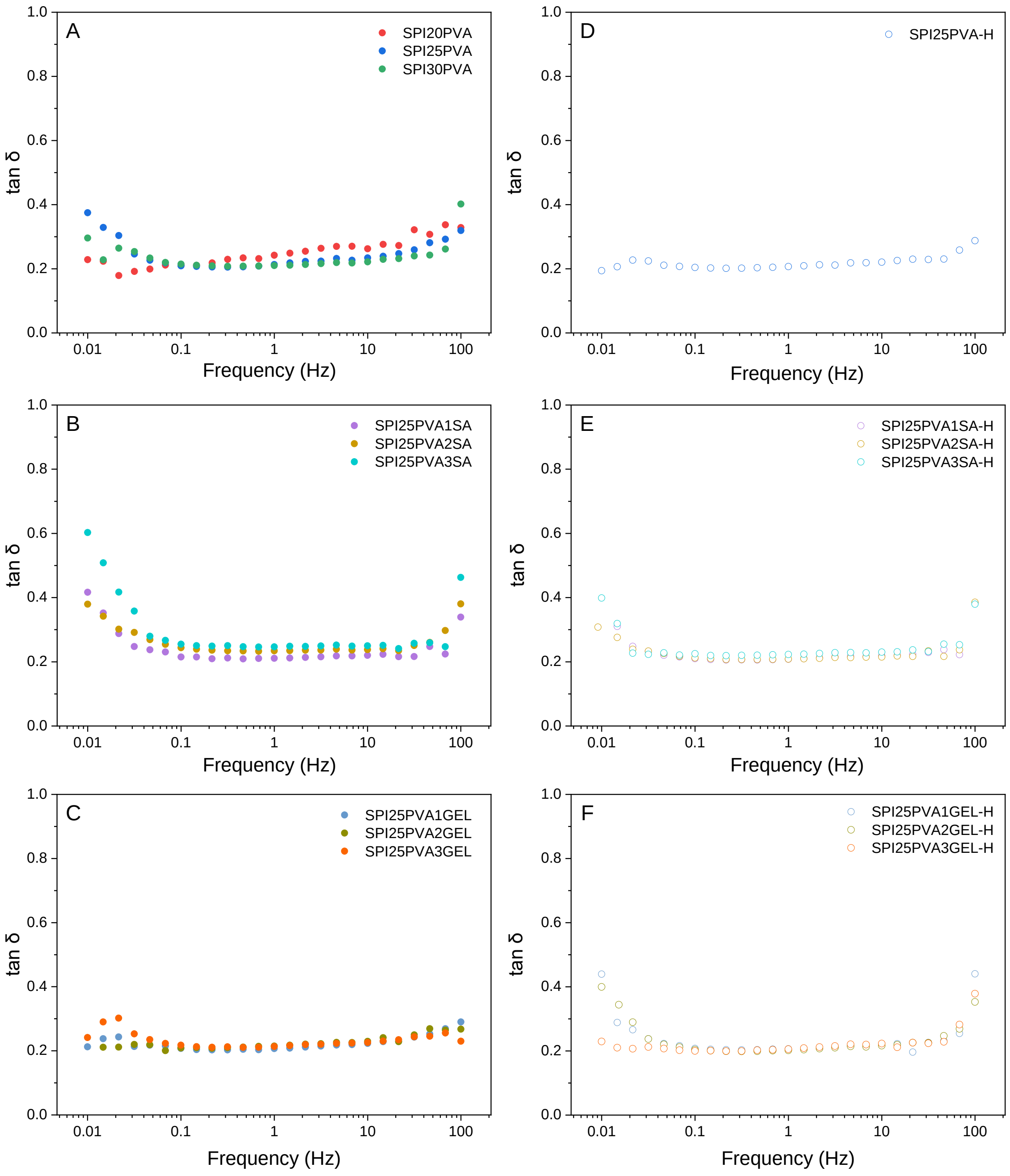

Figure S2. Frequency sweeps of SPI inks with different contents of PVA (20, 25 and 30 wt %) before (A) and after heat treatment (D). Frequency sweeps of SPI25PVA ink with different contents of SA (1, 2, and 3 wt %) before (B) and after heat treatment (E). Frequency sweeps of SPI25PVA ink with different contents of GEL (1, 2, and 3 wt %) before (C) and after heat treatment (F).

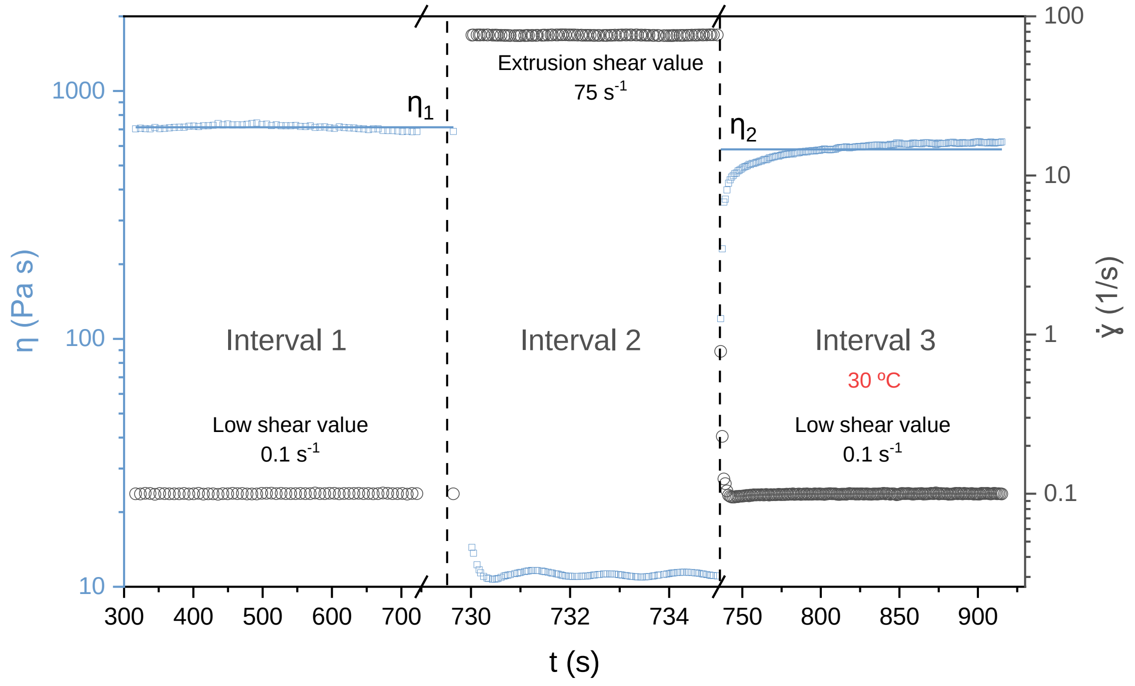

Figure S3. Three interval thixotropy test results for SPI25PVA1GEL ink at 30 ˚C recovery temperature. Mean values of viscosity at interval 1 (η_1) and interval 3 (η_2) were used to calculate the recovery percentage shown in equation (**3**).

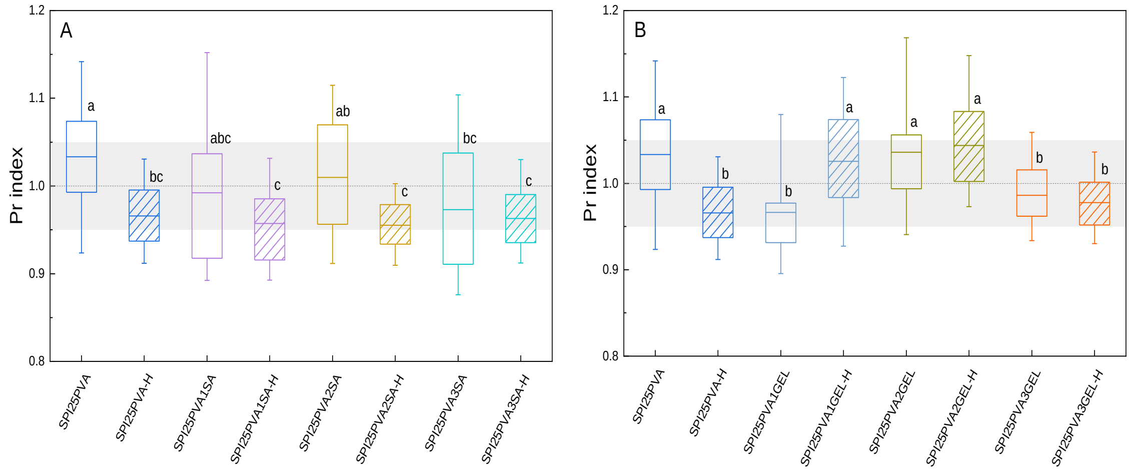

Figure S4. Printability index for SPI25PVA inks with A) different contents of SA (1, 2, and 3 wt %) and B) different contents of GEL (1, 2, and 3 wt %). Line patterns are used for heat-treated (H) inks. The maximum and minimum values are represented with the whiskers. The boxes in the plot represent the 75th and 25th quartiles, indicating the range of the middle 50% of the data. The line inside the box represents the mean value. SPI25PVA ink was used as a control, and statistical significance was calculated for each plotted group (A and B). Means with different letters are significantly different ($p \leq 0.05$) according to Tukey's multiple comparison test.
